# Transient Hip Osteoporosis: Etiopathogenetic, Clinical, and Imaging Approach

**DOI:** 10.31138/mjr.33.2.196

**Published:** 2022-06-30

**Authors:** Nathalie Maisi, Dimitrios Patoulias, Christos Tsagkaris, Michail Tsagatakis, Dimitrios Goules

**Affiliations:** Association of Professional Doctors of Attica, Athens, Greece

**Keywords:** bone edema, transient osteoporosis, etiopathogenesis, MRI, osteonecrosis

## Abstract

Transient Hip Osteoporosis (THO) is a relatively rare, benign, and usually self-limiting disease caused by bone marrow edema, depicted on MRI. It presents with sudden onset of pain or may be asymptomatic. Given the poor coverage of this subject in the relevant international literature and the lack of sufficient understanding of the aetiopathogenic mechanisms underlying this condition, no established diagnostic guidelines are available, leading to frequent under- or overdiagnosis. Therefore, great care should be taken to provide a correct differential diagnosis in order to achieve proper treatment. The international literature from 1990 to 2020 was searched in the PubMed and Google Scholar databases. The keywords: bone marrow edema, transient osteoporosis, osteonecrosis, and aetiopathogenesis were used, and original studies and review articles in English and Greek language were retrieved. Based on evidence provided in the current literature, this article offers a definition and describes in a concise and comprehensive manner the aetiopathogenesis, symptomatology, imaging findings, and differential diagnosis of the disease, in order to provide physicians with a sound knowledge of the condition, necessary to their clinical practice.

## INTRODUCTION

Bone marrow edema (BME), also known as Bone Edema, may represent a primary condition of unknown or uncertain etiology (Primary Bone Edema) or a secondary disease associated with traumatic, inflammatory, degenerative, infectious, autoimmune, malignant, or other conditions. It may also represent an early stage of osteonecrosis with eventual joint collapse, especially in young individuals, which is almost unpredictable.^1–3^

On Magnetic Reasonance Imaging (MRI), bone edema is recognised as an area of focal or diffuse abnormal homogeneous signal with indistinct margins, displaying low intensity on T1 sequences and high intensity on T2 sequences. Its most likely underlying pathological mechanisms are trabecular microfractures, bleeding, and marrow edema with or without disruption of the adjacent cortex or superior articular cartilage. These findings are detected only on MRI and not on conventional radiographs or computed tomography (CT).^4^

Bone edema is caused by damage to the wall of the microvascular network of the bone marrow. This damage initially leads to ischemia followed by reactive hyperemia and vasodilation. It results in edema that increases the intraosseous pressure, causing pain and occasionally necessitating surgical intervention such as core decompression.^5^

Histologicaly, bone needle aspirates show an increase in bone metabolic markers, such as bone specific alkaline phosphatase, osteocalcin, procollagen Type I N-terminal propertide, C-terminal cross-linking telopeptide. It should be noted that these markers are not elevated in the peripheral blood. In bone biopsies, an increased expression of angiogenetic factors (VEGF, CYR61, and CTGF) has also been detected. These factors play a role in the reparative mechanisms, as being inflammatory mediators (cytokines).^5,6^ Due to the lack of consensus regarding classification and nomenclature, our team has recently proposed a simplified and more comprehensive classification of BME or Bone edema Syndrome (BES) offering an improved communication and understanding, which is reported below (**Table 1**).

**Table 1.** Classification of bone oedema syndromes

1. Idiopathic or Primary Oedema Transient (Migratory) Hip Osteoporosis Transient (Migratory) Osteoporosis of Pregnancy
2. Secondary Oedema traumatic, inflammatory, degenerative, infectious, autoimmune, malignant and other conditions
3. Osteonecrosis
4. Osteochondritis (?)

## HISTORY

Transient hip osteoporosis (THO) was first described as a clinical syndrome by Nordin and Roper in 1955.^7^ In 1959, Curtiss and Kincaid reported three cases of unilateral or bilateral hip pain during the third trimester of pregnancy with full recovery to normal after delivery.^8^ In 1988, Wilson described THO as “Transient marrow edema syndrome” with typical MRI finding.^9^ Solomon in 1993 introduced the concept of bone marrow edema with or without osteonecrosis, explaining how the former is associated with bone collapse often requiring surgical intervention, while the latter is transient and self-limiting.^10^ It has been described by different names over the years, but remains poorly understood and is often clinically underestimated. Although interest in bone edema has increased over the past decade, the literature is still poor and consists of studies of small patient groups.

## DEFINITION

Transient Hip Osteoporosis is a relatively rare condition caused by bone marrow edema, which is demonstrated on MRI and presents with sudden pain, or more often, mild progressive worsening pain or even asymptomatically. It is usually a benign self-limiting disease. It still remains a poorly understood condition with limited literature, often clinically underestimated or overestimated and underdiagnosed. Differential diagnosis and clinical experience are required for proper treatment, as there are no guidelines or algorithms yet.

## AETIOPATHOGENESIS

Transient hip osteoporosis has unknown or controversial aetiopathogenesis and multifactorial aetiology. In most cases, no direct or convincing causal events of the disease are detected. Theoretically any insult, such as previous trauma (including fracture, biomechanical disorders), osteoarthritis, inflammatory and autoimmune diseases (arthropathies, enthesopathies, gout), vascular damage (avascular necrosis, algodystrophy), infectious diseases (viral infections, diabetic foot, osteomyelitis), iatrogenic damage (previous surgery or radiotherapy) or neoplasms could be possible causes. Cortisone, smoking, alcoholism, hypothyroidism, reduced testosterone, low vitamin D levels, osteogenesis imperfecta, pregnancy or lactation and other disorders have also been reported to be aetio-logically related, but without any convincing evidence.^11–15^ The aethiopathogenesis has not yet been clearly established, but one of the most widely supported theories has suggested that femoral head vein dysfunction may be the cause of transient hip osteoporosis. This has been favoured by studies of angiography, scintigraphy, and magnetic resonance imaging, claiming that increased intraosseous pressure and venous hypertension of the affected area could be possible pathogenetic mechanisms. Bone biopsy of bone marrow edema showed angiogenesis and fragmentation of fat cells, fibrosis, increased expression of angiogenetic factors and bone metabolic markers. There is no evidence of osteoclastic hyperactivity, but a decrease in hydroxyapatite content of cancellous bone tissue is noted. The elevated bone markers in the bone marrow in combination with the lack of raised bone markers in the peripheral blood are suggestive of high local bone turnover leading to regional reduction in bone density.^16–19^

## CLINICAL PICTURE: PATHOLOGICAL ENTITIES

THO usually presents with two clinical pathological entities: the first (idiopathic THO) usually affects middle-aged men of the 4^th^ and 5^th^ decade of life and the second occurs in young women in the last trimester of pregnancy.^20,21^

### Idiopathic Transient Hip Osteoporosis

It is an idiopathic disease (primary bone odema). It is also called idiopathic transient hip osteoporosis, as the hip is most often involved. It occurs in young and middle-aged men (30–68 years old). It is a condition in which regional bone density decreases. In this phase bone is mechanically weakened, becomes more fragile and the likelihood of fracture increases. It is more common in the hip joint, but can also affect other joints, such as the knee, ankle, or foot. It can involve both hips at the same time or one after the other. It is also common in pregnant women constituting a subcategory of primary edema, and is described below.^20,21^

The causes of THO remain unknown. Various theories have been proposed from time to time, but none of them provides convincing evidence. Disorders of small blood vessels around the femoral head, and hormonal and mechanical abnormalities affecting the bone are reported to be aetiologically related, without clear evidence, however. The clinical symptoms of the disease include sudden onset of pain -without history of injury- in the groin area, in the medial and anterior region of the femur with postero-lateral reflection, and claudication. Pain increases with weight-bearing and decreases with rest. Mild and painful reduction in range of motion is common. Pain usually worsens over time with potential aggravation at sleep or at rest. It mainly causes restriction of patient’s daily activity. Plain films are normal, and MRI demonstrates the typical diagnostic findings of the disease. The disease is usually self-limiting, but its progression to osteonecrosis is unpredictable.^1,22^

### Transient Osteoporosis of Pregnancy

Transient osteoporosis of pregnancy (TOP) is associated with pregnancy (usually the first one) and lactation. It is transient and does not recur in subsequent pregnancies. It usually appears in the last trimester or just after delivery during the immediate postpartum period.^23^ It was first described as a clinical syndrome by Nordin and Roper in 1955.^7^ It is a relatively rare pathological entity. Its incidence is estimated at 0.4 cases per 100,000 women, without taking the large number of undiagnosed cases into account.^24^

The **α**etiopathogenetic mechanism of TOP remains unknown. However, increased body weight and high percentage of adipose tissue, inadequate calcium in-take, smoking, and physical inactivity as well as possible compression of nerves and large blood vessels can be predisposing factors. The diagnosis of TOP presents difficulties related to the young age of patients, the overlap of mild symptomatology of the disease with the normal pain of pregnancy, depression of lactation and possible spinal fractures of pregnancy.^25,26^

The characteristic clinical feature is pain in the hip and medial region of the femur with exacerbation in gait and standing. In the case of spinal fractures (the spinal form of the disease), pain is located in the lower thoracic or lumbar region.^27^

There are also difficulties in assesing bone mass density. Exposure of the fetus to radiation of DXA is a contraindication to this measurement. Furthemore, body weight as well as soft tissue consistency contribute to some difficulties in estimating bone density in pregnant women.^25,28^

The diagnostic method of choice is MRI. Characteristic findings are low signal intensity on T1 sequence and high signal intensity on T2 sequence. In the case of a fracture, a thin band of low intensity signal is shown in the area of edema while a low intensity signal on T1 sequence and a high intensity signal on T2 sequence are seen in the surrounding area representing edema.^15^

### Imaging Findings

Various imaging modalities are performed such as plain X-rays, CT, ultrasound, scintigraphy, and MRI. On MRI, bone edema is detected with high sensitivity, even 48 hours after the onset of symptoms,^29,30^ as opposed to avascular necrosis, which usually presents bone edema in later stages of the disease. The edema is located in the femoral head and neck, while it can sometimes extend to the intertrochanteric band. MRI can also demonstrate possible subchondral fractures. The absence of subchondral focal lesions is characteristic of transient osteoporosis.

MRI is the only method that takes advantage of the presence of protons to produce images. These protons are usually found in water molecules and the presence of a pathological signal on MRI is due to the disturbance of proton concentration per unit volume of water in the bone marrow.^31^

The normal magnetic signal of the bone marrow parallels that of subcutaneous fat, being high on T1-weighted sequences and intermediate to low on T2-weighted fat suppressed sequences.^32,33^

In contrast, bone edema due to increased water content in the bone marrow, displays low or intermediate intensity signal on T1-weighted sequences (higher than muscle or intervertebral discs) and high intensity signal on T2-weighted sequences including STIR imaging.^22^

**Figure 1.**
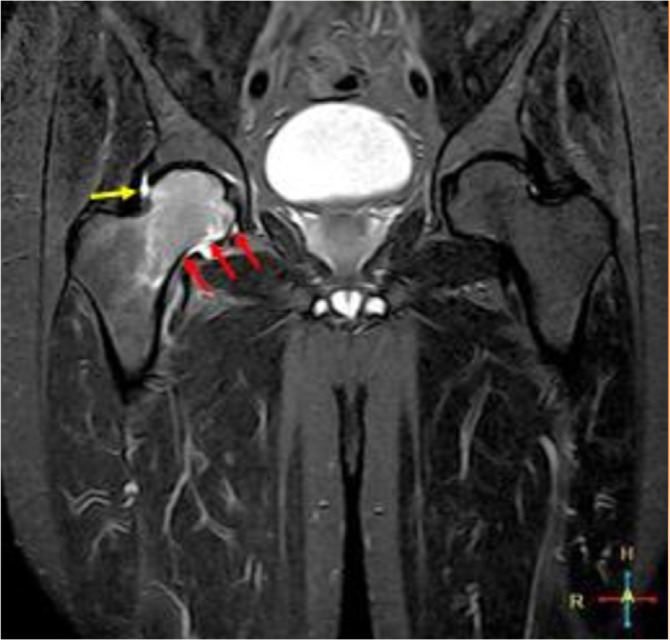
Transient hip osteoporosis (T2 STIR sequence at the coronal level): Extensive disturbance of magnetic signal intensity of the right femoral head and neck with increased signal intensity on T2 STIR sequence, corresponding to bone edema (red arrows). The contour of the femoral head is kept normal. A small amount of fluid collection in the joint is seen (yellow arrow).

Following intravenous contrast medium administration, bone edema shows increased medium uptake, compared to normal bone marrow. Other features of bone edema are the relative homogeneity of the pathological signal and its indistinct margins at the interface with normal bone marrow (**Figure 1**). ^15, 22^

Magnetic resonance imaging is able to demonstrate an irregular band of low magnetic signal on all sequences potentially associated with a stress fracture. It is controversial whether bone edema is due to a stress fracture or vice versa.^32^

**Figure 2.**
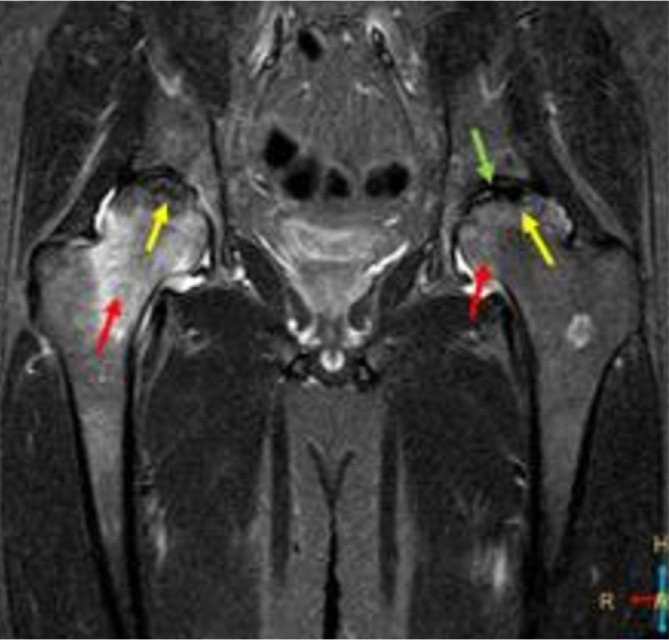
Avascular necrosis (T2 STIR sequence at the coronal level: **Grade III** Avascular necrosis of the right femoral head: Pathological focus on subchondral surface of the bone, which is surrounded by a double linear halo (yellow arrow) and an extensive bone edema (red arrow). **Grade IV** Avascular necrosis of the left femoral head. Pathological focus on the subchondral surface of the bone, which is surrounded by a double linear halo (yellow arrow) and a milder bone edema (red arrow). Deformity of the articular surface of the bone (green arrow).

In femoral head osteonecrosis, T1 imaging usually reveals a hypointense band of damage in the anterior and superior femoral head, while the “double-line sign” is often seen on T2 imaging, on which a hyperintense signal of automatic repair is present adjacent to necrotic subchondral bone (**Figure 2**).^33^

When MRI is non-diagnostic, other imaging methods can be performed, such as plain films, CT scans, and scintigraphy. Radiographs may complement MRI, leading to a more precise differential diagnosis.^32,34^

Reduced regional bone density (regional osteoporosis) on plain radiographs constitutes a late finding of bone edema syndromes, and may persist for some time after the symptoms have resolved. CT scan is unable to depict bone edema, but is used to rule out bone lesions associated with bone edema, such as osteoid osteoma.^28,35^ Scintigraphy plays a limited role as long as magnetic resonance imaging is available. However, it has the advantage of evaluating both the affected area and the rest of the skeleton in some patients to exclude metastatic neoplasms.^36^

### Differential diagnosis

The differential diagnosis includes osteonecrosis, neoplasms, arthritis, and infections. Avascular necrosis is the first condition to be excluded, as it has a similar clinical and imaging picture. Magnetic resonance imaging may help in distinguishing them. In many instances, but unfortunately not always, images of avascular necrosis display a subchondral lesion (semicrescent morphology), bordered by a serpentine line of low-intensity signal on T1 sequence, the double line sign (an outer hypointense line representing sclerosis and an inner hyperintense line corresponding to granulomatous tissue) with surrounding edema on T2 sequence and may be accompanied by distortion of the femoral head. Following contrast medium administration, the pathological lesions may show filling defects, signal change in subchondral regions, and focal alteration (**Figure 2**).

The absence of subchondral focal lesions, the delayed enhancement of the pathological bone marrow on post contrast images, and the detection of a subchondral band of normal bone marrow, without bone edema, are MRI findings which represent possible transient osteoporosis (**Figure 1**).

MRI also detects stress fractures, both in avascular necrosis and in transient hip osteoporosis, as a filling defect and a fracture line in the former, as well as an irregular low signal line in the latter.^21^ Infections, tumor lesions, and rheumatoid arthritis can be excluded by both clinical and imaging findings. Transient osteoporosis could mimic enchondromata on X-rays as an area of radiolucency, but lacks central calcifications.^38^

## CONCLUSIONS

Transient hip osteoporosis is a rare disease of unknown aetiology with several risk factors. We maintain that it presents with two pathological entities, the idiopathic form (most common) and that of pregnancy. It is usually a benign, self-limiting disease. The imaging findings play a key role in the diagnosis, but also in the differential diagnosis, as there is a possibility of eventual osteonecrosis. Unfortunately, however, MRI findings are often inconclusive in the differential diagnosis between THO and early avascular necrosis. This review is a useful tool for physicians dealing with musculoskeletal disorders.
